# Mapping mitochondrial aging

**DOI:** 10.7554/eLife.105191

**Published:** 2024-12-20

**Authors:** Alessandro Bitto

**Affiliations:** 1 Department of Laboratory Medicine and Pathology, University of Washington Medical Center Seattle United States

**Keywords:** mitochondria, respiration atlas, sex, aging, cellular respiration, Mouse

## Abstract

Measuring mitochondrial respiration in frozen tissue samples provides the first comprehensive atlas of how aging affects mitochondrial function in mice.

**Related research article** Sarver DC, Saqib M, Chen F, Wong GW. 2024. Mitochondrial respiration atlas reveals differential changes in mitochondrial function across sex and age. *eLife* **13**:RP96926. doi: 10.7554/eLife.96926.

There are multiple theories as to why animals grow old and die. One of the first theories of aging blames free radicals for our eventual demise (Harman, 2003). Free radicals are unstable molecules that can damage cellular components, and they are thought to originate from mitochondria – the organelles responsible for harnessing energy from nutrients.

Mitochondria use a metabolic process called respiration to store energy from nutrients in the form of a molecule known as ATP, which powers many essential cellular processes. This mechanism mostly takes place in the mitochondrial inner membrane, which houses protein complexes that make up an electron transport chain – the final stage of respiration responsible for driving the bulk of ATP production. It is generally accepted that mitochondria become less active in aging animals and that their dysfunction is a key contributor to the aging process (López-Otín et al., 2023).

Markers of mitochondrial dysfunction have been seen in a range of aging model organisms, from yeast to nematode worms (Hughes and Gottschling, 2012; Berry et al., 2023), as well as in aged human cells in culture (Lerner et al., 2013). In mice, inducing mitochondrial dysfunction causes premature aging (Trifunovic et al., 2004; Kujoth et al., 2005), and interventions that protect or restore mitochondrial function have successfully delayed aging in several models (Schriner et al., 2005; Berry et al., 2023). However, the extent and magnitude of mitochondrial decline with age and its impact on aging more generally is not fully understood.

A device called a ‘respirometer’ can be used to measure mitochondrial activity by detecting how much oxygen these organelles are consuming. However, until recently, this approach could only be applied to freshly isolated mitochondria obtained from mammalian tissues through a long and laborious process, making them difficult to study in large numbers. This limitation has prevented comprehensive analyses of mitochondrial respiration in mammalian tissues. Now, in eLife, William Wong and colleagues from Johns Hopkins University – including Dylan Sarver as first author – report the first large-scale analysis of mammalian mitochondrial respiration and how this process changes with aging (Sarver et al., 2024).

Using a recently developed protocol for respiratory analysis of frozen tissue samples (Acin-Perez et al., 2020), Sarver et al. measured a proxy of mitochondrial respiration in over 1000 samples from a large cohort of young and old mice of both sexes. This included tissues with reportedly high mitochondrial activity, such as certain brain regions, several skeletal muscles, the heart, and the kidneys. The samples also included metabolic tissues like the liver or pancreas, as well as sections of the gastrointestinal tract, the skin and the eyes.

Due to the process of freezing and thawing, the mitochondria in the samples were not intact and therefore could not be isolated. As a result, Sarver et al. measured mitochondrial respiration at three different sites on the electron transport chain in cellular extracts enriched with mitochondrial membranes. The proteins making up this chain are likely to remain relatively stable in mitochondria whose membrane integrity has been lost, which allows measurements that indicate the maximum capacity of the mitochondria to produce ATP to be taken.

Analyzing the differences between old and young animals revealed a net decline in mitochondrial activity in most tissues with age, most notably in samples from the brain and metabolic tissues. These results are consistent with our current understanding of the energetic demands of various tissues and how they decline over time. They also support previous findings which demonstrated age-related changes in mitochondrial gene expression and electric activity (Schaum et al., 2020).

Intriguingly, in older animals, respiration increased in some tissues with high-energy demand, such as the heart and skeletal muscles, which is potentially at odds with the observation that these organs perform less well with age. Analyzing differences between samples from males and females also revealed that age has a much larger effect on mitochondrial activity across all tissues than sex.

Although the findings do not reveal the mechanisms behind the observed changes, Sarver et al. offer a roadmap for future studies aimed at better understanding the role of mitochondrial respiration in aging. Notably, as the team acknowledged, the techniques used to generate this ‘respiration atlas’ cannot recapitulate the full extent of mitochondrial physiology. Their method does not account for important physiological variables that might impact respiration, such as nutrient and oxygen availability, transport of reagents and products across mitochondrial membranes, and how proteins in the electron transport chain are organized. At the moment, these can only be assessed in intact mitochondria or tissue preparations.

Furthermore, because the technique measured the maximum capacity of the electron transport chain to produce energy in compromised mitochondria, the values may not correspond to the actual level of respiration in intact mitochondria or tissue slices, nor necessarily reflect how much of this respiration actually produces ATP. Regardless, the convenience of the method and the ability to analyze samples on a much larger scale will be a driving force behind studying the broader impact of various interventions on respiration.

Geroscience, which aims to understand how the aging drives disease and develop strategies to slow it (Kennedy et al., 2014), has identified several genetic, pharmacological, and environmental interventions that can increase lifespan and reduce, if not reverse, the pace of aging in model organisms. Some, such as compounds called rapamycin or urolithin A, are known to affect mitochondrial function in certain contexts, while others have no clear effect on mitochondria. Regardless, a full-scale picture of how such treatments influence respiration systemically is still missing. Given that samples from treated animals are widely available to the scientific community through tissue repositories, applying the methods outlined by Sarver et al. offers an ideal starting point for studying the true impact of these longevity strategies on mitochondrial function. Such studies are likely to generate new and surprising hypotheses in geroscience research and contribute to our understanding of how critical mitochondrial function really is for our overall health and well-being.
